# Rumination as a Mediator Between Intolerance of Uncertainty and Online Health Anxiety, Moderated by Medical History

**DOI:** 10.3390/diseases14050171

**Published:** 2026-05-13

**Authors:** Mălina-Andreea Apostol, Simona Trifu, Andrei-Gabriel Zanfir, Amelia-Damiana Trifu

**Affiliations:** 1 Faculty of Psychology and Educational Studies, University of Bucharest, 050663 Bucharest, Romania; malina-andreea.apostol61@s.fpse.unibuc.ro; 2 Department of Clinical Neurosciences, “Carol Davila” University of Medicine and Pharmacy, 020021 Bucharest, Romania; 3 Doctoral School, “Carol Davila” University of Medicine and Pharmacy, 020021 Bucharest, Romania; andrei-gabriel.zanfir@drd.umfcd.ro; 4 Medico-Military Institute, “Carol Davila” University of Medicine and Pharmacy, 020021 Bucharest, Romania

**Keywords:** cyberchondria and health anxiety, intolerance of uncertainty in mental health, rumination and online health-information seeking, psychological predictors of cyberchondria, health anxiety and digital health behavior

## Abstract

Objective: We examined the psychological mechanisms underlying cyberchondria by testing whether rumination mediates the association between intolerance of uncertainty and cyberchondria and whether this indirect effect is moderated by prior medical experiences and perceived access to healthcare. Methods and Measures: A cross-sectional design was employed with a non-clinical sample of 96 Romanian adults. Participants completed validated self-report measures of intolerance of uncertainty (IUS-12), rumination (Ruminative Responses Scale), and cyberchondria (Cyberchondria Severity Scale). Additional items assessed medical history and perceived access to healthcare. Moderated mediation analyses with bootstrapped confidence intervals were conducted, controlling for relevant sociodemographic variables. Results: Higher intolerance of uncertainty was associated with higher cyberchondria both directly and indirectly through rumination, which accounted for more than half of the total effect. The rumination–cyberchondria association, and the indirect effect of IU, were significantly stronger among individuals who had experienced a recent acute medical episode, whereas chronic illness did not significantly moderate this pathway. Cyberchondria levels were lowest among participants reporting very good access to healthcare. Conclusions: Cyberchondria appears to arise from the interaction of intolerance of uncertainty, ruminative thinking, and contextual health experiences. Targeting rumination and uncertainty tolerance may be particularly important following acute medical events.

## 1. Introduction

Cyberchondria is defined as excessive or repeated online searching for health-related information that is accompanied by increased distress or health anxiety and persists despite interference with functioning and negative consequences [1]. Unlike adaptive health-information seeking, cyberchondria is characterized by compulsive search patterns, difficulty disengaging from searches, and a counterintuitive amplification of anxiety despite accumulated information [2]. Meta analytic evidence demonstrates a strong association between cyberchondria and health anxiety (r = 0.63), indicating that cyberchondria constitutes a distinct construct related to but not subsumed by health anxiety, obsessive–compulsive symptoms, and anxiety sensitivity [1].

Cyberchondria can be further conceptualized as a maladaptive online behavioral manifestation of health anxiety, emerging when individuals attempt to reduce uncertainty about their health through repeated internet-based symptom checking. While traditional health anxiety involves excessive worry about having or developing a serious illness, cyberchondria reflects the digital expression of these concerns, shaped by the accessibility, volume, and ambiguity of online medical information. Empirical findings show that individuals with elevated health anxiety are more likely to engage in frequent and prolonged online health searches and to experience increased distress during and after these searches [3]. Meta-analytic evidence further supports a robust association between health anxiety and cyberchondria [4]. However, cyberchondria is not reducible to health anxiety alone. Systematic reviews [1] indicate that it is a multidimensional construct, also linked to obsessive–compulsive tendencies, intolerance of uncertainty, anxiety sensitivity, and problematic internet use. Thus, cyberchondria is best understood not as a synonym for health anxiety, but as a distinct, technology-mediated behavioral pattern through which health anxiety is amplified and maintained in the digital environment.

The paradoxical nature of cyberchondria, wherein information seeking intensifies rather than alleviates health concerns, reflects underlying meta-cognitive processes. According to an integrative theoretical model, health anxiety activates information needs; when individuals encounter ambiguous or contradictory medical information online, those with dysfunctional metacognitive beliefs (e.g., beliefs that health-related thoughts are uncontrollable) develop compulsive searching behaviors characteristic of cyberchondria [5]. Individuals with elevated health anxiety demonstrate increased engagement with online medical content, access to forums and self-diagnostic tools, and consequently greater exposure to medical misinformation [2,6]. Recent systematic reviews characterize cyberchondria as a transdiagnostic compulsive behavioral syndrome associated with health anxiety, hypochondriasis, obsessive–compulsive disorder, and problematic internet use [1]. The psychological mechanisms maintaining cyberchondria include low self-esteem, anxiety sensitivity, intolerance of uncertainty, pain catastrophizing, and dysfunctional metacognitive beliefs. A key mechanism involves safety-seeking behavior: individuals ritualistically search for health information to reduce anxiety, continuing until they achieve a subjective threshold of reassurance or certainty regarding their health status [7]. The clinical consequences of cyberchondria extend beyond psychological distress. Cyberchondria severity predicts significantly increased healthcare utilization, including visits to primary care physicians, specialists, hospital admissions, and emergency department use, independent of baseline health anxiety levels [3]. Furthermore, cyberchondria severity predicts a reduced uptake of preventive health behaviors such as COVID-19 vaccination [8,9]. Individuals with cyberchondria report sleep disturbances, reduced physical activity, and erosion of trust in healthcare providers, all contributing to poor health outcomes and reduced treatment adherence [10]. A concerning aspect is the role of information quality and digital literacy. The internet provides unprecedented access to medical information, yet substantial proportions of online health content are inaccurate, contradictory, or deliberately misleading [11]. The phenomenon of “apparent consensus”, defined as the identical health claims that propagate across multiple websites, creating a false impression of scientific legitimacy, amplifies the persuasiveness of misinformation. Individuals with limited health literacy are particularly vulnerable to these information hazards [12].

### 1.1. Intolerance of Uncertainty (IU)

IU denotes the propensity to react negatively, emotionally, cognitively, and behaviorally to ambiguous situations [13]. It functions as a transdiagnostic risk factor for generalized anxiety, obsessive–compulsive disorder, social phobia, and depression [14]. Dugas et al. showed IU to be the strongest discriminator between patients with generalized anxiety disorder and non-clinical controls [15]; Shihata et al. highlighted both trait- and disorder-specific IU dimensions as robust predictors of clinical symptoms and maladaptive metacognitive beliefs [16].

### 1.2. IU and Cyberchondria

Persons with a high IU are more likely to engage in compulsive online medical searches in an attempt to reduce perceived health uncertainty, yet this strategy amplifies worry and fuels rumination and checking cycles [17]. IU correlates with cyberchondria severity and problematic smartphone use, linking rapid information access with compulsive behaviors [18]. Individuals high in IU favor authoritative-appearing web sources while simultaneously mistrusting health professionals, further reinforcing self-verification [19]. A 2023 breast cancer survivor study reported that IU significantly predicts health anxiety (*p* < 0.001) and that this effect is partially mediated by compulsive information seeking and low cognitive flexibility (*p* < 0.001) [20].

### 1.3. Rumination

Rumination is repetitive, passive focus on the causes and consequences of emotional stress without problem-solving intent, thereby sustaining a negative affect and increasing the risk of anxiety and depression [21,22]. It disrupts adaptive information processing and executive functions, intensifying negative bias and rigid self-focus [23]. Clinically, rumination is linked to major depression, generalized anxiety, and obsessive–compulsive disorder and predicts greater symptom severity and relapse [24]. It also heightens health anxiety, promoting catastrophic interpretations of somatic cues. It is demonstrated that rumination directly raises health anxiety and indirectly does so via reduced serenity, a calming positive affect [25]. Sansom Daly et al. found that ruminators recall past medical problems in greater detail but generate vague, illness-focused future projections, diminishing cognitive specificity [26].

### 1.4. IU, Rumination, and Cyberchondria

High IU can trigger rumination, which in turn drives compulsive online health searching and escalates health anxiety. Pirooz identified rumination as a significant mediator between IU, anxiety sensitivity, and health anxiety; individuals who ruminate are especially vulnerable to health-related anxiety under uncertainty [27]. Błachnio et al. reported that rumination mediates the link between the threat appraisal of stress and compulsive medical information seeking [28]. Yook et al. showed that, in major depressive disorder, rumination fully mediates the relationship between IU and depressive severity, indicating that intolerance of uncertainty fuels persistent negative self-referential thinking [29].

### 1.5. Psychological Variables and Medical Conditions

In chronic illness, rumination amplifies emotional distress and mediates the relationship between negative illness perceptions and symptoms [30]; researchers observed this pattern in rheumatoid arthritis and systemic lupus patients, where rumination correlated with negative emotions and fatigue [31]. A meta-analysis of 30 prospective studies confirmed strong associations between rumination and emotional distress across diverse chronic conditions, and baseline rumination predicted later worsening of affective symptoms [32]. Recovery from acute medical events can be protracted; Gardner and Moallef described three stages of psychological recovery after severe acute respiratory syndrome, noting persistent anxiety, depression, and post-traumatic stress that may last years, underscoring the need for long-term psychological support [33].

The current literature largely examines direct IU–cyberchondria links, neglects intermediate mechanisms, conflates state and trait anxiety, and relies on student samples, limiting external validity [1,34,35]. The present study therefore adopts a moderated mediation framework to test whether rumination mediates the IU–cyberchondria relationship and whether this indirect effect is conditioned by prior medical experiences and perceived access to health services.

### 1.6. Study Aims and Hypotheses

The present study aims to clarify how intolerance of uncertainty (IU), rumination, and prior medical experiences jointly shape vulnerability to cyberchondria, extending earlier findings that IU alone accounts for a substantial proportion of cyberchondria variance and that rumination mediates this relationship, with the indirect effect being amplified by acute medical episodes. Specifically, the investigation tests the following hypotheses:

**H1.**  *Higher levels of intolerance of uncertainty will predict higher levels of cyberchondria.*

**H2.**  *Rumination will mediate the relationship between intolerance of uncertainty and cyberchondria.*

**H3.**  *Prior medical experiences will moderate the relationship between rumination and cyberchondria, such that the association will be stronger among individuals with recent acute medical experiences compared to those without such experiences or with chronic conditions.*

**H4.**  *The indirect effect of intolerance of uncertainty on cyberchondria through rumination will be conditional on prior medical experiences, being stronger at higher levels of medical vulnerability (particularly following acute medical episodes).*

**H5.**  *Cyberchondria levels will differ significantly according to participants’ perceived access to medical services.*

## 2. Materials and Methods

### 2.1. Participants

Participants were recruited using a snowball sampling strategy, with social media platforms serving as the primary channel for questionnaire distribution. The survey link was initially posted in a set of university-related online groups, groups of students and young professionals, as well as on personal pages, then shared further by respondents who were encouraged to forward it to individuals from their networks who met the eligibility criteria. Data were collected using an online questionnaire created and administered through Google Forms between 15 May 2025 and 30 June 2025. Recruitment took place exclusively online, without direct face to face contact between researchers and participants.

The target population consisted of students and recent university graduates. Inclusion criteria were minimum age of 18 years, current residence in Romania, being enrolled in or having recently completed a university program, sufficient command of the Romanian language to understand the items, access to the internet, and the ability to complete an online questionnaire independently. Exclusion criteria were age below 18 years, self-reported severe cognitive impairment or current severe psychiatric disorder that could interfere with the capacity to provide informed consent, extremely short completion time suggesting random responding, and duplicate submissions identified by identical time stamps and response patterns.

Information regarding current severe psychiatric disorders was obtained through a self-report item asking participants whether they had received a diagnosis of a severe mental disorder in the last year that significantly interfered with daily functioning. Respondents who endorsed this option were thanked for their interest and not allowed to proceed to the main questionnaire.

Before data collection, an a priori power analysis was conducted using G*Power version 3.1, test family F tests, linear multiple regression, fixed model, R^2^ deviation from zero for a model with up to five predictors, corresponding to the most complex path in the moderated mediation model. Assuming a small to medium effect size of f^2^ = 0.08, an alpha level of 0.05, and desired statistical power of 0.80, the analysis indicated a minimum required sample size of 166 participants for detecting the effect of the predictors on the outcome within the regression framework. To account for possible exclusions due to missing data or low-quality responses, the target sample size was set at approximately 100 completed questionnaires. The final analyzable sample of 96 participants met this a priori criterion and provided adequate power for detecting small to medium effects on the main regression paths. Even if the study is underpowered for moderation, the results will clearly indicate that the specific moderation effect is shown to be statistically significant. Power for detecting interaction effects in moderated mediation is generally lower than for main effects, and the results regarding moderation should therefore be interpreted with appropriate caution.

Before accessing the questionnaire, all participants read an information sheet describing the aims of the study, the anonymous and confidential nature of data processing, and their right to withdraw at any point by closing the browser window without any consequences. Informed consent was recorded electronically by requiring participants to actively indicate agreement before proceeding to the first item. Because some participants were students at the authors’ home institution, particular care was taken to minimize any perception of coercion. Participation was voluntary and unpaid, was not linked to course grades or academic evaluation, and the invitation was distributed via general online announcements rather than direct approaches from teaching staff. No information that could identify individual participants was available to teaching personnel.

Data were downloaded from Google Forms, anonymized by removing any potentially identifying information, and stored on password-protected institutional servers. Only the authors directly involved in data analysis had access to the dataset.

The survey link was accessed by 178 individuals. Of these, 124 started the questionnaire and 108 completed all items. Twelve cases were excluded from the analyses. Six cases were removed because they failed the attention check item, three cases were removed because they displayed highly patterned responding, for example, the same response option selected across almost all items, and three cases were removed because the questionnaire completion time was less than five minutes, which was defined a priori as implausibly short based on pilot testing and the observed distribution of completion times. A minimum completion time threshold of five minutes was defined a priori based on pilot testing and inspection of the distribution of completion times. During pilot administration, the questionnaire required approximately 12–15 min to complete when answered attentively. Completion times below five minutes were therefore considered insufficient for careful reading and responding to all items, suggesting random or inattentive responding. The final sample therefore consisted of 96 participants. Because sociodemographic items were presented at the beginning of the questionnaire, limited information on age and gender was available for some partial responders; inspection of these distributions did not suggest marked differences between completers and non-completers, although formal statistical comparisons were not conducted due to the small number of partial responders with available data.

The final sample included 96 adults residing in Romania. Of these, 45 participants were male (46.9%) and 51 were female (53.1%). Age ranged from 18 to 57 years, with a mean of 30.02 years and a standard deviation of 9.12. Regarding educational attainment, 19 participants had completed secondary education and were currently enrolled in university studies (19.8%), 42 held an undergraduate degree (43.8%), and 35 had completed postgraduate studies (36.4%). The majority lived in urban areas, 83 participants (86.5%), while 13 participants reported residing in rural settings (13.5%).

Perceived access to healthcare services varied across the sample. No participant rated their access as poor. Thirty-four participants described access as satisfactory, indicating some difficulties that were generally manageable (35.4%). Forty-three participants rated access as good, meaning that services were generally available with minor delays (44.8%), and 18 participants reported very good access, defined as rapid access without notable difficulties (18.8%). Perceived access was coded on a four-level ordinal scale from 1, satisfactory, to 4, very good, and was used as a four-level factor in the analysis of variance that compared cyberchondria across categories of access.

With respect to the frequency of online health-information seeking, 20 participants reported engaging in this behavior rarely or never (20.8%), 53 searched for health information online approximately once per month (55.2%), 18 did so weekly (18.8%), and 5 reported very frequent online health information searches, defined as several times per week or more (5.2%). This variable was coded on a four-level ordinal scale and entered as a covariate in sensitivity analyses to test the robustness of the main findings.

Regarding medical history, 49 participants reported no diagnosis of a chronic illness and no significant acute medical episode in the previous year (51.0%). In contrast, 24 participants reported at least one significant acute episode in the past year but no chronic condition (25.0%), and 23 participants reported having a chronic medical diagnosis (24.0%). Medical history was assessed with the question “Have you ever been diagnosed with a chronic illness, or have you experienced a significant acute medical episode in the last year?” This was followed by explanatory examples. Chronic illness was defined as a long-term condition that requires ongoing treatment or medical monitoring, such as diabetes, asthma, autoimmune diseases, cardiovascular diseases, or neurological conditions. An acute episode was defined as a medical problem with sudden onset, short duration, and intense symptoms, for example, severe COVID-19 infection, major surgery, or a severe infection. For the analyses, prior medical experiences were coded on a three-level ordinal scale, 0 for no diagnosis and no significant acute episode, 1 for at least one significant acute episode but no chronic illness, and 2 for the presence of a chronic illness. In the moderated mediation models, this ordinal variable was treated as a continuous moderator, reflecting an ordered increase in the severity and chronicity of medical experiences. This assumes a monotonic relationship between the level of medical history and cyberchondria, an assumption supported by the observed pattern of means across the three categories. Sensitivity analyses with dummy coded indicators using the no diagnosis group as reference yielded a similar pattern of results.

### 2.2. Measures

#### 2.2.1. Intolerance of Uncertainty

Intolerance of uncertainty was assessed with the 12 item Intolerance of Uncertainty Scale, IUS 12, short form of the original instrument developed by Carleton and colleagues. The validated Romanian version was used, in line with published adaptation guidelines [36]. The scale comprises 12 statements describing reactions to uncertain situations, rated on a five-point Likert scale from 1, not at all characteristic of me, to 5, entirely characteristic of me. There are no reverse scored items. The total score is the sum of all items, with higher scores indicating greater intolerance of uncertainty [14].

The IUS 12 yields two subscales, prospective anxiety items 1 to 7 and inhibitory anxiety items 8 to 12. In the present study, only the total score was entered in the main models, to limit model complexity and to capture the general tendency to perceive uncertainty as aversive. Subscale scores were computed and reported descriptively. Internal consistency was good in this sample, Cronbach’s alpha of 0.89 for the total score, consistent with previous reports [14,37].

#### 2.2.2. Assessment of Rumination

Rumination was measured with the 22 item Ruminative Responses Scale, RRS, originally developed within the response styles framework for depression [38,39]. The validated Romanian version proposed by Lazăr and colleagues was used [38]. Items describe cognitive self-focus, repetitive thinking, and passive evaluation of one emotional state. Responses are given on a four-point Likert scale from 1, never, to 4, always.

Total RRS scores were obtained by summing all 22 items, with higher scores reflecting a stronger tendency to respond to negative mood through rumination. Although the RRS can be scored on three subscales, Depression—12 items, Reflection—5 items, and Brooding—5 items, only the total score was included in the regression and PROCESS analyses to avoid interpretative overlap between the Depression and Brooding subscales reported in earlier work [38,39]. Subscale scores were calculated and summarized descriptively to characterize the ruminative profile of the sample. Internal consistency was excellent in the present data; Cronbach’s alpha was 0.95 for the total RRS.

#### 2.2.3. Cyberchondria

Cyberchondria was measured with the 33-item Cyberchondria Severity Scale (CSS) assessing frequency and impact of online health searches [40,41]. Because no validated Romanian version existed, items were translated through a forward–backward procedure by bilingual psychologists, discrepancies were resolved by consensus, and the prefinal version was piloted on 15 adults.

Items describe behaviors, cognitions, and emotions related to excessive health information seeking, rated on a five-point Likert scale (1 = never, 5 = always). The CSS yields one total score and five subscales: Compulsion, Distress, Excessiveness, Reassurance Seeking, and Distrust of Medical Professionals [40,41,42]. Only the total score was used in analyses; higher scores indicate higher cyberchondria.

Internal consistency was excellent (α = 0.94 total; subscales = 0.86–0.93), comparable to previous validations [40,41,42]. Exploratory factors and convergent-validity analyses with intolerance of uncertainty and rumination are presented in the results section.

### 2.3. Sociodemographic and Clinical Variables

The questionnaire also included items assessing age, gender, educational level, study program or field, area of residence, perceived access to healthcare, frequency of online health-information seeking, and medical history. These variables were used to describe the sample and, in the case of age, gender, educational level, and online search frequency, were included as covariates in the main regression models (Table 1).

### 2.4. Procedure and Data Analysis

The study used an observational correlational cross-sectional design based on a non-probabilistic online sample obtained through snowball recruitment among students and related populations. After reading the information sheet and providing informed consent, participants completed the measures in a fixed order to minimize missing data. The questionnaire began with sociodemographic items, followed by the medical history questions, then the IUS 12, RRS, and CSS. On average, completion required approximately fifteen minutes. The questionnaire was configured in Google Forms so that essentially all scale items required a response before proceeding, which contributed to the very low rate of missing data.

One attention check item was embedded in the questionnaire. This item instructed participants to select a specific response option, for example, to choose a particular number, in order to verify that they were reading the questions carefully. Participants who failed this item were excluded from the analyses, as described above.

Data were exported from Google Forms into IBM SPSS Statistics, version 24.0. Data screening included inspection of missing values, ranges, and distributional characteristics of the main variables. Cases with more than 10% missing data, failed attention checks, or implausibly short completion times were removed listwise. For the remaining cases, item-level missing data were minimal, being under 5%. Given this low proportion and the absence of obvious systematic patterns in missingness when cross tabulated with key sociodemographic variables, no imputation procedures were applied. The small amount of missing data was handled using pairwise deletion in descriptive statistics and listwise deletion in regression and analysis of variance models.

Descriptive statistics were computed for all variables, including means, standard deviations, and Pearson correlations for the continuous measures and frequency distributions for categorical variables. Internal consistency for the IUS 12, RRS, and CSS was estimated using Cronbach’s alpha. All continuous predictor variables were mean centered before inclusion in interaction terms in order to reduce multicollinearity and facilitate interpretation of regression coefficients. The dependent variable, cyberchondria, was used in its original metric. Standardized coefficients, R^2^, and change in R^2^ for the regression models are reported in the Results section.

The primary analytic model was a moderated mediation in which intolerance of uncertainty served as the predictor, rumination as the mediator, and cyberchondria as the outcome variable. Prior medical experiences, coded as described above, were entered as a moderator of the association between rumination and cyberchondria. Age, gender, educational level and frequency of online health-information seeking were included as covariates in all models to control for potential confounding effects.

Hypothesized relationships were tested using the PROCESS macro for SPSS, version 4.2. Model 4 was used to test the simple mediation of rumination in the association between intolerance of uncertainty and cyberchondria. Model 1 was used to examine the moderation of the rumination and cyberchondria association by prior medical experiences. Model 14 was used to test the full moderated mediation model. The significance level was set at alpha equal to 0.05 and two tailed. Indirect and conditional effects were estimated using the bootstrap method with five thousand resamples and 95% confidence intervals. Effects were considered statistically significant when the corresponding confidence intervals did not include zero. All PROCESS analyses used heteroscedasticity consistent standard errors of type HC3.

Assumptions for linear regression were examined by inspecting residual plots and normal probability plots. The normality of residuals was evaluated using the Shapiro–Wilk test and by visual inspection of histograms and Q–Q plots. Homoscedasticity was checked by plotting standardized residuals against standardized predicted values. Multicollinearity was assessed using tolerance values and variance inflation factors, which indicated no serious problems. Potential influential cases were examined using Cook distance and leverage values; no cases exceeded conventional thresholds, so all retained cases were included in the final analyses, and no variable transformations were applied.

For exploratory analyses on differences in cyberchondria across levels of perceived access to healthcare services, one way analysis of variance was conducted. Homogeneity of variances was evaluated using Levene’s test. When this assumption was violated, Welch-corrected statistics and Games–Howell post hoc tests were reported. For the analysis of variance, effect sizes were indexed by partial eta squared. No formal corrections for multiple testing were applied, as the analyses were guided by hypotheses specified a priori embedded in a single coherent moderated mediation framework rather than by a large number of independent exploratory comparisons.

## 3. Results

The analyses followed a prespecified sequence: descriptive statistics and intercorrelations among intolerance of uncertainty, rumination and cyberchondria, examination of the simple association between intolerance of uncertainty and cyberchondria, testing of a mediation model with rumination as the mediator, testing of a moderated mediation model with prior medical experiences as the moderator, and evaluation of differences in cyberchondria across levels of perceived access to healthcare.

### 3.1. Descriptive Statistics and Correlations

Intolerance of uncertainty (IUS-12), rumination (RRS-22), and cyberchondria (CSS-33) all showed substantial variability in the sample. Intolerance of uncertainty had a mean of 32.30 (SD 10.44, observed range 12–51), rumination had a mean of 47.06 (SD 13.72, range 22–86), and cyberchondria had a mean of 82.22 (SD 23.80, range 34–129). Internal consistency was good to excellent for all three instruments (Cronbach’s α between 0.89 and 0.95).

Pearson correlations indicated positive associations among all three constructs. Intolerance of uncertainty was strongly associated with rumination (r = 0.73, *p* < 0.001) and moderately to strongly associated with cyberchondria (r = 0.59, *p* < 0.001), while rumination was also positively associated with cyberchondria (r = 0.64, *p* < 0.001) (Table 2). These correlations are of a moderate to large magnitude and consistent with the hypothesized model in which a higher intolerance of uncertainty and more ruminative responding co-occur with higher levels of cyberchondria.

### 3.2. Association Between Intolerance of Uncertainty and Cyberchondria (H1)

A simple linear regression analysis was conducted to examine the association between the intolerance of uncertainty and cyberchondria. The model was statistically significant, *F*(1, 94) = 49.96, *p* < 0.001, with the intolerance of uncertainty accounting for 34.7% of the variance in cyberchondria (R^2^ = 0.347, adjusted R^2^ = 0.340). The Durbin–Watson statistic (1.62) indicated no problematic autocorrelation of residuals. The examination of residual plots suggested approximate normality and homoscedasticity, and no influential outliers were detected.

The unstandardized regression coefficient indicated that a higher intolerance of uncertainty was associated with higher cyberchondria (B = 1.34, SE 0.19, t = 7.07, *p* < 0.001, and 95% CI 0.97–1.72). This corresponds to a standardized coefficient β = 0.59, indicating a large effect in conventional terms. The resulting regression equation was as follows: cyberchondria = 38.86 + 1.34 × (intolerance of uncertainty) (Table 3).

These results are consistent with hypothesis (H1), whereby individuals who report greater difficulty tolerating uncertainty also report higher levels of cyberchondria.

### 3.3. Mediation by Rumination (H2)

To test whether the association between the intolerance of uncertainty and cyberchondria is statistically mediated by rumination, a mediation analysis was conducted using Model 4 of the PROCESS macro, with the intolerance of uncertainty (IUS) as the predictor, rumination (RRS) as the mediator, and cyberchondria (CSS) as the outcome.

Intolerance of uncertainty was positively associated with rumination (path a: B = 0.97, SE 0.08, t = 12.12, *p* < 0.001, and 95% CI 0.81–1.13). In turn, rumination was positively associated with cyberchondria when controlling for intolerance of uncertainty (path b: B = 0.76, SE 0.16, t = 4.80, *p* < 0.001, and 95% CI 0.45–1.07). The total effect of intolerance of uncertainty on cyberchondria was statistically significant (path c: B = 1.34, SE 0.19, t = 7.07, *p* < 0.001, and 95% CI 0.97–1.72).

When rumination was added to the model, the direct effect of intolerance of uncertainty on cyberchondria (path c) remained statistically significant but was reduced in magnitude (B = 0.61, SE 0.26, t = 2.32, *p* = 0.022, and 95% CI 0.09–1.13). The indirect effect of intolerance of uncertainty on cyberchondria through rumination was B = 0.73 (bootstrapped SE 0.20, 95% CI 0.35–1.11). The standardized indirect effect was β = 0.32 (95% CI 0.16–0.48). Approximately 54.5% of the total association between the intolerance of uncertainty and cyberchondria was carried through rumination, with 45.5% remaining as a direct effect (Table 4).

Because the confidence interval for the indirect effect did not include zero, the pattern of results is consistent with hypothesis (H2) and with a mediation model in which a higher intolerance of uncertainty is associated with higher cyberchondria in part via higher levels of rumination. Given the cross-sectional design, these findings should be interpreted as evidence of statistical mediation rather than definitive proof of temporal or causal ordering.

### 3.4. Moderation and Moderated Mediation by Prior Medical Experiences (H3, H4)

Prior medical experiences were coded as a three-level variable: (1) no chronic illness and no significant acute medical episode in the previous year reference group, (2) at least one significant acute episode but no chronic illness, and (3) chronic medical illness. For PROCESS analyses, two dummy variables were created: W1, acute episode versus reference; W2, chronic illness versus reference.

#### 3.4.1. Moderation of the Rumination–Cyberchondria Association, H3

The moderation model with cyberchondria as the outcome, rumination as the predictor, and prior medical experiences as the moderator was significant, with R = 0.70, R^2^ = 0.50, *F*5,90 = 17.70, and *p* less than 0.001; see Table 5A.

Across the sample, higher rumination was associated with higher cyberchondria. This association was stronger in participants with a recent acute episode, as indicated by the significant RRS × W1 interaction, whereas the RRS × W2 interaction, chronic illness versus reference, was not significant. Conditional effects showed that rumination predicted cyberchondria in all three medical history groups, and the slope was steepest in the acute episode group, intermediate in the chronic illness group, and smallest in the no episode group; see Table 5. These findings support H3 and suggest that recent acute medical events accentuate the impact of rumination on cyberchondria.

#### 3.4.2. Moderated Mediation H4

**Figure 1:**
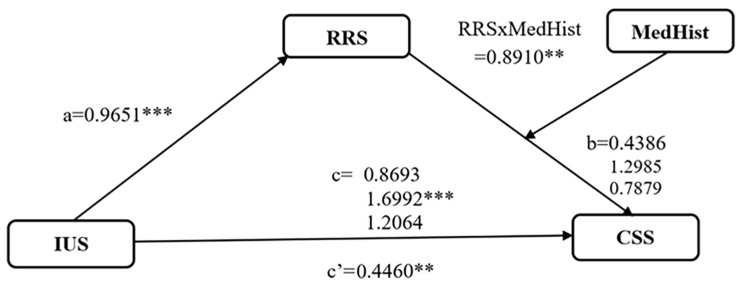
Moderated mediation model with intolerance of uncertainty (IUS) as predictor, rumination (RRS) as mediator, cyberchondria (CSS) as outcome, and prior medical experiences (MedHist) as moderator of the RRS–CSS path. ** *p* < 0.01; *** *p* < 0.001.

The moderated mediation model PROCESS Model 14 tested whether the indirect association between the intolerance of uncertainty and cyberchondria via rumination varies by prior medical experiences; see Figure 1 and Table 5B. Intolerance of uncertainty remained positively associated with rumination path a, and the rumination–cyberchondria path b was again moderated by acute, but not chronic, medical episodes, with the RRS × W1 term being significant and the RRS × W2 term being non-significant.

The conditional indirect effects of intolerance of uncertainty on cyberchondria through rumination were small to moderate in participants without acute episodes or chronic illness, largest in those with a recent acute episode, and intermediate in those with chronic illness. The index of moderated mediation was significant for the contrast acute episode versus reference but not for chronic illness versus reference; see Table 5.

### 3.5. Differences in Cyberchondria by Perceived Access to Healthcare (H5)

A one-way analysis of variance was conducted to evaluate whether levels of cyberchondria differed across categories of perceived access to healthcare. Participants were classified into three groups: satisfactory access (*N* = 35), good access (*N* = 43), and very good access (*N* = 18).

The omnibus ANOVA indicated a significant effect of perceived access on cyberchondria, *F*(2, 93) = 4.27, *p* = 0.017, with a partial η^2^ of approximately 0.08, corresponding to a small to medium effect. Because Levene’s test of homogeneity of variances was borderline (*p* = 0.056), Welch’s robust test was also examined and confirmed significant between-group differences, *F*(2, 50.20) = 6.26, *p* = 0.004.

Descriptively, the highest mean level of cyberchondria was observed in participants reporting good access to healthcare (M = 88.07, SD 21.59), followed by those reporting satisfactory access (M = 80.69, SD 24.87), while the lowest levels were observed in participants reporting very good access (M = 69.22, SD 17.62). Games–Howell post hoc tests indicated that cyberchondria scores were significantly higher in the “good access” group than in the “very good access” group (mean difference = 18.85, 95% CI 3.46–34.24, and *p* = 0.012). Differences involving the “satisfactory access” group did not reach statistical significance (Table 6).

These results provide partial support for hypothesis (H5), suggesting that cyberchondria varies with perceived access to healthcare and tends to be lower among individuals who perceive their access to be very good.

## 4. Discussion

The present study examined how intolerance of uncertainty, rumination, and prior medical experiences jointly contribute to cyberchondria in a Romanian sample. The findings provide conceptual, methodological, and clinical contributions to the literature on maladaptive online health information seeking. Consistent with prior research, intolerance of uncertainty emerged as a robust predictor of cyberchondria, supporting evidence that individuals who struggle with ambiguity and doubt are more likely to engage in excessive online medical searching [43,44]. The present study extends this literature by identifying psychological mechanisms through which the intolerance of uncertainty may exert its influence, as well as contextual conditions that may intensify this pathway.

Intolerance of uncertainty was associated with cyberchondria both directly and indirectly through heightened rumination. This finding aligns with cognitive–behavioral models suggesting that the fear of uncertainty promotes perseverative negative thinking [45,46]. Although rumination has previously been identified as a mediator in cyberchondria-related pathways, such as stress appraisal [28] and information overload [47], fewer studies have examined rumination as the explanatory mechanism linking the intolerance of uncertainty to cyberchondria, particularly in relation to contextual health variables. By testing whether this indirect pathway is moderated by prior medical experiences, the present study clarifies not only whether the intolerance of uncertainty is associated with cyberchondria through rumination but also for whom this pathway may be strongest. These findings suggest that the intolerance of uncertainty may translate into maladaptive online reassurance seeking through a ruminative cycle that heightens the perceived threat and increases repeated symptom checking. This pathway expands prior models that have emphasized either direct cognitive effects or alternative mediators, such as fear of illness [48,49]. Demonstrating that rumination partially carries the influence of intolerance of uncertainty highlights a clinically modifiable mechanism that may be important for reducing maladaptive digital health behaviors.

Beyond identifying rumination as a mechanism, the present study shows that the strength of this ruminative pathway differs according to prior medical experiences. Specifically, individuals who had recently experienced acute medical episodes showed a significantly stronger association between rumination and cyberchondria than those with chronic illness or no significant medical history. This finding is consistent with theoretical accounts suggesting that acute health events heighten vigilance toward bodily sensations and increase reliance on reassurance-seeking behaviors [50]. However, prior empirical studies have generally examined medical history as a correlational or categorical background factor [51,52] rather than as a moderator of psychological pathways. Thus, the current moderated mediation model provides novel evidence that medical experiences may function as contextual amplifiers of cognitive processes, particularly rumination, that contribute to cyberchondria. The distinction between recent acute episodes and chronic conditions further suggests that salient, emotionally charged health events may sensitize individuals to uncertainty more strongly than ongoing but familiar medical conditions.

The PROCESS Model 14 analysis demonstrated that the indirect effect of intolerance of uncertainty on cyberchondria through rumination is conditional, becoming substantially stronger following an acute health event. This analytic configuration, which links the intolerance of uncertainty as the predictor, rumination as the mediator, cyberchondria as the outcome, and medical history as the moderator, has rarely been applied in cyberchondria research. Prior complex models have typically positioned the intolerance of uncertainty as a mediator between personality traits and cyberchondria [53] or emphasized emotional intermediaries such as the fear of COVID-19 [54]. The current structure therefore advances the field by demonstrating that medical experiences may influence not just the onset of rumination but the consequences of rumination, making it a more potent catalyst for online health anxiety and checking behaviors.

Another important contribution of this study relates to perceived access to healthcare. Although scholars have argued that barriers to healthcare access may encourage greater reliance on online health-information seeking [55,56], few empirical studies have tested this association directly. The present findings showed that cyberchondria scores were lowest among participants who rated their healthcare access as very good and were significantly higher even among those reporting good access. This gradient suggests that cyberchondria may arise partly from subtle variations in the perceived availability of professional reassurance rather than only from overt system-level barriers. Improving provider communication, appointment accessibility, and responsiveness may therefore help mitigate excessive online health searching.

Methodologically, the present study provides preliminary evidence regarding the reliability and expected correlational patterns of the Romanian version of the Cyberchondria Severity Scale (CSS-33). The scale demonstrated high internal consistency and showed theoretically consistent associations with the intolerance of uncertainty and rumination. However, no formal psychometric validation was conducted, as the study did not examine factor structure, measurement invariance, or criterion validity. Further research is needed to establish the full psychometric properties of the instrument in Romanian samples. While several validated versions exist in Spanish, Italian, Polish, and Arabic [57,58], little published work has documented the properties of the CSS in Romanian populations. Our findings—including strong internal consistency and expected associations with IU and rumination—support the usability of the CSS-33 in this cultural context.

The use of a psychologically informed sample also provides insight into mechanisms that operate even among individuals with greater-than-average familiarity with psychological concepts. Notably, intolerance of uncertainty, rumination, and medical history remained significant predictors even after controlling for self-reported frequency of online health-information seeking. This suggests that these associations are not merely artifacts of baseline patterns of digital health behavior but instead reflect distinct psychological and contextual contributions to cyberchondria.

The study found that intolerance to uncertainty accounts for 34% of the variance in cyberchondria (*p* < 0.001). We explicitly note that this is a large effect. For a large correlational effect, an *N* (number of subjects) of 96 provides excellent statistical power (well over 95%). The relationships between intolerance of uncertainty and rumination (b = 0.9651, *p* < 0.001), as well as rumination and cyberchondria (b = 0.7604, *p* < 0.001), are highly statistically significant. When *p*-values are this small (*p* < 0.001), it indicates that the signal-to-noise ratio in the data is very strong. The analysis of moderation in this research indicates that the interaction between rumination and previous medical experiences significantly predicts cyberchondria (b = 0.891, *p* = 0.0196). Because the *p*-value (0.0196) is below the standard alpha threshold of 0.05, the study successfully detected the effect. The argument that “the study is underpowered for moderation” becomes moot once the specific moderation effect is shown to be statistically significant.

Overall, the findings have several theoretical and clinical implications. First, they support conceptualizations of cyberchondria as a multistage process that begins with intolerance of uncertainty, unfolds through ruminative thinking, and culminates in compulsive digital reassurance seeking. Second, they identify populations that may require greater psychological support, particularly individuals recovering from acute medical episodes who appear especially vulnerable to rumination-driven cyberchondria. Third, interventions targeting uncertainty tolerance, rumination such as rumination-focused cognitive behavioral therapy, and illness-related memory and interpretation may be especially beneficial [59,60]. Moreover, strengthening the perceived access to healthcare may reduce reliance on online information as a substitute for professional reassurance.

### 4.1. Limitations and Future Directions

Several methodological limitations should be considered.

First, the sample was relatively small and recruited through non-probabilistic, online snowball procedures, and all participants were students or graduates. Although the study had adequate power for medium effects in the tested models, the modest sample size reduces the precision of estimates and limits the ability to detect smaller effects. The educational and age profile, with a concentration in early to mid-adulthood, further constrains external validity. This population may possess prior knowledge of the constructs under investigation (e.g., intolerance of uncertainty, rumination, and health anxiety), potentially increasing the likelihood of hypothesis awareness. Such familiarity may introduce demand characteristics or response biases, as participants might consciously or unconsciously align their responses with perceived study expectations. Age-related differences in health anxiety and related constructs have been documented, with anxiety-related vulnerabilities showing distinct patterns in older adults [61]. Replication in a larger, more demographically diverse community and clinical samples is needed.

Second, all variables were measured by self-report in a single cross-sectional assessment. Medical history, including the presence of chronic diagnoses and significant acute episodes, could not be verified against medical records. Although the acute episode item was accompanied by examples, it still allowed for considerable subjectivity in judging severity. Research on post-infectious conditions suggests that subjective illness perception is strongly influenced by experienced symptoms and may diverge from objective clinical indicators [62]. Therefore, the present results reflect perceived, rather than clinically verified, medical experiences. Future research could combine self-report measures with documented diagnoses, symptom scales, and independent clinical information, allowing researchers to examine convergence and divergence between subjective and objective indicators.

Third, medical experiences were operationalized in broad categories that did not distinguish between diagnostic groups, symptom burden, or treatment complexity. Different conditions may have distinct psychological correlates and trajectories. The current approach prioritized the perceived presence of chronic disease or recent acute episodes as psychologically salient events. Subsequent studies could examine whether specific disease categories or symptom patterns differentially interact with the intolerance of uncertainty, rumination, and cyberchondria.

Fourth, the design was cross-sectional and correlational. The moderated mediation model was specified a priori and grounded in existing theory; however, temporal precedence cannot be inferred. Longitudinal designs are needed to test whether the intolerance of uncertainty prospectively predicts changes in rumination and cyberchondria and whether acute medical events amplify these pathways over time. Experimental and micro-longitudinal work could further clarify how day-to-day fluctuations in uncertainty, mood, and bodily sensations drive online health-information seeking.

Fifth, several potentially important covariates were not assessed or controlled, including general health anxiety, depressive symptoms, obsessive–compulsive symptoms, and specific metacognitive beliefs. Prior studies indicate that cyberchondria is related to, but not identical to, health anxiety and obsessive–compulsive phenomena and may be best conceptualized as a digital compulsive pattern anchored in these broader vulnerability dimensions [4,63,64,65]. Including standard measures of health anxiety and related constructs, such as the Health Anxiety Inventory [64], alongside the Cyberchondria Severity Scale would allow for a more precise estimation of unique and shared variance.

### 4.2. Theoretical and Practical Implications

Despite these limitations, the findings have several implications for theory and practice. Conceptually, the data support models that place the intolerance of uncertainty and repetitive negative thinking at the center of cyberchondria. The association between these constructs and cyberchondria aligns with the broader literature on transdiagnostic cognitive processes in anxiety and related conditions, as well as with proposals that cyberchondria represents a digital expression of such processes in the health domain [44,45,63,66]. The results are also compatible with metacognitive formulations, in which beliefs about the necessity, usefulness, and danger of health-related thinking, combined with internet affordances, maintain patterns of checking and reassurance seeking that are difficult to disengage from [43,67].

From a clinical perspective, the findings suggest that the assessment of cyberchondria may benefit from routine evaluation of intolerance of uncertainty, rumination, and online health-information seeking, particularly among individuals who present with high health anxiety or frequent reassurance seeking. Interventions targeting the intolerance of uncertainty and rumination, such as cognitive behavioral protocols and approaches focused on reducing repetitive negative thinking, have shown beneficial effects in related conditions [65,66,68]. Adaptations of these interventions that explicitly address online searching episodes, including their short-term relief and long-term costs, may be particularly relevant for cyberchondria. Metacognitive therapy, which directly addresses beliefs about worry and thought control, also appears conceptually well matched to the cognitive profile identified here [43,67].

The moderating effect of recent acute medical experiences highlights a potential window for preventive intervention. Psychoeducational and brief psychological interventions delivered in the aftermath of acute medical episodes could normalize uncertainty, clarify typical trajectories of recovery and provide strategies for managing rumination and online health-information seeking. Such interventions may reduce the likelihood that acute events lead to persistent and distressing patterns of digital checking.

The association between very positive perceptions of healthcare access and lower levels of cyberchondria underscores the relevance of system-level and educational factors. Research on health literacy and the evaluation of online information indicates that individuals with low health literacy are more likely to have difficulty judging the quality of web-based health resources and to experience greater distress after searching [4,61,69]. Efforts to improve health literacy, increase transparency about access to care, and provide clear guidance toward credible online resources may complement individual-level interventions and contribute to reducing cyberchondria at the population level.

## 5. Conclusions

The present study provides new insights into the psychological and experiential factors that shape cyberchondria in a non-clinical sample with relatively high literacy. By integrating the intolerance of uncertainty, rumination, and prior medical experiences into a unified moderated mediation model, the study demonstrates that cyberchondria does not arise from a single cognitive vulnerability but from the dynamic interplay of multiple mechanisms. Intolerance of uncertainty emerged as a central predictor, consistent with theoretical accounts that position discomfort with ambiguity as a driving force behind compulsive and distress-amplifying health-related searches. A substantial proportion of this effect was explained through rumination, highlighting repetitive negative thinking as a key process through which uncertainty is translated into heightened online reassurance seeking.

A further novel contribution lies in the identification of recent acute medical experiences as an amplifying factor. Individuals who had recently experienced a significant acute episode showed a markedly stronger link between rumination and cyberchondria, suggesting that salient and emotionally charged health events may heighten cognitive reactivity and increase the perceived need to seek information online. In contrast, chronic illness did not significantly alter the strength of the rumination–cyberchondria pathway, likely reflecting the stabilizing effect of established illness management routines. This distinction underscores the importance of considering the type and recency of medical experiences when assessing vulnerability to cyberchondria.

The finding that cyberchondria varied with perceived access to healthcare, with the lowest levels observed among those reporting very good access, further reinforces the role of structural and contextual factors. While cyberchondria is fundamentally a cognitive–emotional process, it is shaped by individuals’ confidence in their ability to obtain timely, trustworthy, and satisfactory professional care. These results suggest that enhancing perceptions of access and quality of care may indirectly reduce the reliance on digital reassurance seeking.

The findings support a multidimensional model of cyberchondria in which cognitive vulnerability to uncertainty, ruminative processing styles, recent health events, and perceived system-level access converge to influence online health behaviors. This integrative perspective has practical implications for assessment and intervention. Addressing intolerance of uncertainty and repetitive negative thinking through established cognitive–behavioral or metacognitive techniques may reduce susceptibility to distress-amplifying digital searches. For individuals recovering from acute medical episodes, early psychoeducation on uncertainty, bodily vigilance, and online information-seeking patterns may serve as a preventive strategy. At a broader level, improving health literacy and promoting transparent communication about healthcare access and pathways could mitigate some of the system-level factors that contribute to cyberchondria.

The study also contributes methodologically by offering preliminary psychometric support for the Romanian version of the Cyberchondria Severity Scale, enabling future cross-cultural work in this area. Although limited by its cross-sectional design, modest sample size, and reliance on self-reported medical histories, the study provides a coherent empirical basis for future longitudinal and experimental research. Clarifying how cyberchondria unfolds over time, and how acute health stressors interact with cognitive vulnerabilities, will be an essential step toward developing targeted, scalable interventions. In an era of unprecedented access to online health information, understanding these mechanisms is critical for promoting adaptive digital engagement and supporting more effective healthcare utilization.

## Figures and Tables

**Table 1:** Sociodemographic and clinical characteristics of the sample (*N* = 96).

Variable	Category	*N*	%	M (SD)
Age (years)	-	96	-	30.02 (9.12)
Gender	Male	45	46.9%	
	Female	51	53.1%	
Educational level	Secondary education (currently enrolled in university)	19	19.8%	
	Undergraduate degree	42	43.8%	
	Postgraduate studies	35	36.4%	
Study field	Psychology/related mental health fields	96	100%	
Area of residence	Urban	83	86.5%	
	Rural	13	13.5%	
Medical history	No chronic illness; no significant acute episode in past year	49	51.0%	
	≥1 significant acute episode; no chronic illness	24	25.0%	
	Chronic medical diagnosis	23	24.0%	
Perceived access to healthcare	Satisfactory	35	35.4%	
	Good	43	44.8%	
	Very good	18	18.8%	
	Poor	0	0.0%	
Frequency of online health-information seeking	Rarely/never	20	20.8%	
	About once per month	53	55.2%	
	Weekly	18	18.8%	
	Very frequent (several times per week or more)	5	5.2%	

**Table 2:** Descriptive statistics, reliability, and correlations for main study variables (*N* = 96).

Variable	Possible Range	Observed Range	M	SD	α	1	2	3
1. Intolerance of uncertainty (IUS-12)	12–60	12–51	32.30	10.44	0.89	-		
2. Rumination (RRS-22)	22–88	22–86	47.06	13.72	0.95	0.73 ***	-	
3. Cyberchondria (CSS-33)	33–165	34–129	82.22	23.80	0.94	0.59 ***	0.64 ***	-

Note: α = Cronbach’s α. *** *p* < 0.001.

**Table 3:** Linear regression of cyberchondria (CSS) on intolerance of uncertainty (IUS) (*N* = 96).

	B	SE B	β	t	*p*	95% CI for B (Lower, Upper)	R	R^2^	*F*(1,94)	*p* (Model)
Model summary	-	-	-	-	-	-	0.589	0.347	49.96	<0.001
Constant	38.86	6.44	-	6.03	<0.001	26.06, 51.65	-	-	-	-
IUS-12 (total)	1.34	0.19	0.59	7.07	<0.001	0.97, 1.72	-	-	-	-

Note: dependent variable: cyberchondria (CSS total score).

**Table 4:** Mediation analysis of the relationship between intolerance of uncertainty and cyberchondria through rumination (*N* = 96).

Path/Effect	B	SE	t/z	*p*	95% CI Lower	95% CI Upper
a: IUS → RRS	0.97	0.08	12.12	<0.001	0.81	1.13
b: RRS → CSS (controlling IUS)	0.76	0.16	4.80	<0.001	0.45	1.07
c: IUS → CSS (total effect)	1.34	0.19	7.07	<0.001	0.97	1.72
c′: IUS → CSS (direct effect)	0.61	0.26	2.32	0.022	0.09	1.13
Indirect effect (a × b)	0.73	0.20	-	-	0.35	1.11
Standardized indirect effect	0.32	0.08	-	-	0.16	0.48

Note: Indirect effect and its confidence interval obtained using 5000 bootstrap samples (PROCESS Model 4).

**Table 5:** Moderation and moderated mediation by prior medical experiences (MedHist) (n = 96).

(A) Moderation Model: Rumination (RRS) and Medical History Predicting Cyberchondria (CSS)						
Predictor	B	SE	t	*p*	95% CI Lower	95% CI Upper
Constant	45.50	9.15	4.97	<0.001	27.31	63.68
RRS (centered)	0.65	0.20	3.25	0.002	0.25	1.05
RRS × W1 (acute episode)	1.03	0.37	2.79	0.006	0.30	1.77
RRS × W2 (chronic illness)	0.43	0.30	1.42	0.158	−0.17	1.02
Model summary: R = 0.70, R^2^ = 0.50, *F*(5, 90) = 17.70, and *p* < 0.001.						
**(B) Conditional Indirect Effects and Indices of Moderated Mediation (IUS → RRS → CSS)**						
**MedHist Level**			**Indirect Effect (B)**		**95% CI Lower**	**95% CI Upper**
1. No acute episode/no chronic illness			0.44		0.04	0.84
2. Acute episode			1.30		0.57	2.02
3. Chronic illness			0.79		0.31	1.34
Index of moderated mediation			B		95% CI lower	95% CI upper
W1: acute episode vs. reference			0.86		0.17	1.56
W2: chronic illness vs. reference			0.35		−0.15	0.91

Note: W1 = dummy contrast acute episode vs. no acute episode/chronic illness; W2 = dummy contrast chronic illness vs. no acute episode/chronic illness. Indirect effects and indices of moderated mediation estimated using PROCESS Model 14 with 5000 bootstrap samples.

**Table 6:** Cyberchondria (CSS) by perceived access to healthcare (n = 96).

Perceived Access to Healthcare	*N*	M CSS	SD CSS
Satisfactory	35	80.69	24.87
Good	43	88.07	21.59
Very good	18	69.22	17.62
**Omnibus tests:** **ANOVA: *F*(2, 93) = 4.27, *p* = 0.017, and partial η^2^ ≈ 0.08.** **Welch’s test: *F*(2, 50.20) = 6.26, *p* = 0.004.** **Games–Howell post hoc comparisons:** **Good vs. very good: mean difference = 18.85, 95% CI 3.46–34.24, and *p* = 0.012.** **Satisfactory vs. good: ns.** **Satisfactory vs. very good: ns.**			

## Data Availability

Data is contained within the article.

## Abbreviations

The following abbreviations are used in this manuscript:

ANOVA	Analysis of variance
CBT	Cognitive-behavioral therapy
CI	Confidence interval
CSS	Cyberchondria Severity Scale
EFA	Exploratory factor analysis
IU	Intolerance of uncertainty
IUS	Intolerance of Uncertainty Scale
IUS-12	12-item Intolerance of Uncertainty Scale
MedHist	Prior medical experiences
M	Mean
*N*	Sample size
PAF	Principal axis factoring
PROCESS	PROCESS macro for SPSS
RRS	Ruminative Responses Scale
SD	Standard deviation
SE	Standard error
SPSS	IBM SPSS Statistics
